# Monotropein Induced Ferroptosis to Alleviate the Progression of Hepatocellular Carcinoma via Regulating Nrf2/HO‐1/GPX4 Axis

**DOI:** 10.1002/kjm2.70034

**Published:** 2025-05-29

**Authors:** Bing Shi, Yan‐Ping Li, Zhuo Gan, Pan Chen

**Affiliations:** ^1^ Department of Nuclear Medicine The First Hospital of Hunan University of Chinese Medicine Changsha China

**Keywords:** anti‐tumor, ferroptosis, hepatocellular carcinoma, monotropein, Nrf2/HO‐1/GPX4 axis

## Abstract

Hepatocellular carcinoma (HCC) exhibits a high global morbidity rate and ranks as the fourth leading cause of cancer‐related mortality worldwide. In response to the urgent need for effective HCC treatments, naturally occurring, botanical‐driven compounds have gained increasing attention. Notably, the anti‐tumor properties of some compounds might be linked to the induction of ferroptosis. The present study aimed to evaluate the capacity of Monotropein (Mon) to induce ferroptosis in HCC and elucidate its underlying mechanisms. First, Mon was found to play an anti‐tumor role in HCC cells by inhibiting cell proliferation and invasion, elevated the expression of E‐cadherin, and decreased N‐cadherin and Vimentin expression. Furthermore, Mon activated ferroptosis in HCC cells, characterized by elevated levels of Fe^2+^, reactive oxygen species (ROS), and malondialdehyde (MDA), alongside a reduction in glutathione (GSH) content and downregulation of nuclear factor E2‐related factor 2 (Nrf2), heme oxygenase‐1 (HO‐1), and glutathione peroxidase 4 (Gpx‐4). These in vitro findings were confirmed by in vivo tumorigenicity experiments. With regard to the mechanism, the suppression of Nrf2 signaling played a significant role in facilitating ferroptosis induced by Mon, ultimately slowing down the progression of HCC cells. In conclusion, this study revealed that Mon suppressed the progression of HCC both in vitro and in vivo, which was closely associated with ferroptosis induction via inhibiting Nrf2 signaling. These results suggest that Mon represents a promising alternative for HCC treatment.

## 1 Introduction

For a long time, the prevalence of liver cancer has been recognized as a significant public health concern [1, 2]. Hepatocellular carcinoma (HCC) exhibits a high morbidity rate globally and ranks as the fourth leading cause of tumor‐related mortality worldwide [3, 4]. Despite considerable advancements in therapeutics, many cases of HCC continue to be diagnosed at advanced stages. Traditionally, patients with advanced HCC have faced limited treatment options and poor prognoses [5, 6]. Furthermore, the limitations associated with currently available therapeutic agents are well documented; these include drug toxicity, drug resistance, and prohibitive costs. Consequently, there is a pressing need to identify safer and more cost‐effective agents that could serve as potential therapies to improve the prognosis of HCC.

In recent years, there has been a growing interest in the exploration of naturally occurring, botanical‐driven compounds as potential modalities, which offer multi‐targeting and safer therapeutic options with low toxicity [7, 8]. Monotropein (Mon) is an active iridoid glycoside compound isolated from the roots of Morinda officinalis F.C. How. Mon exhibits multiple pharmacological bioactivities, including anti‐inflammatory, antioxidant, and anti‐apoptotic effects [9]. Mon has been reported with therapeutic effects in various noncancerous disease models. Li et al. [10] have reported that Mon alleviates the progression of osteoarthritis in mice by attenuating apoptosis and pyroptosis in chondrocytes, indicating an anti‐osteoporosis role. Gong et al. [11] have found that Mon mitigates sepsis‐evoked acute lung injury by inhibiting apoptosis, inflammation and fibrosis; Qiang et al. [12] have shown that Mon protects against sepsis‐induced colonic injury through the induction of autophagy, suggesting a lung or colonic protective role against sepsis. Regarding cancer research, Lu et al. [13] have indicated that Mon inhibits colitis‐associated cancer through modulation of macrophage polarization; Quan et al. have demonstrated that Mon exerts its anti‐tumor properties primarily by arresting the cell cycle and inducing cell apoptosis in colorectal cancer [14]. However, the potential effects of Mon on HCC still need to be investigated.

A substantial body of research has demonstrated that the anti‐tumor effects of naturally occurring compounds may be specifically associated with their capacity to induce ferroptosis, a unique programmed cell death featured with iron‐dependent lipid peroxidation [15, 16]. Ferroptosis is initiated by the accumulation of free iron ions, followed by the elevation of reactive oxygen species (ROS) and lipid peroxidation, ultimately resulting in iron‐dependent cell death in tumor cells [17]. Lian et al. [18] have shown that puerarin functions as an anticancer compound in colorectal cancer cells by promoting ferroptosis. Hu et al. [19] have reported that tiliroside induces ferroptosis to relieve the triple‐negative breast cancer progression. Han et al. [20] have discovered that the flavonoid compound Pt3R5G suppresses colon cancer cell proliferation via facilitating ferroptosis. The above studies imply that the induction of ferroptosis serves as an endogenous anticancer strategy; and in this context, some naturally occurring compounds have been explored as ferroptosis inducers in tumor cells.

The induction of ferroptosis has been reported as a potential strategy for HCC treatment [21]. Therefore, we wondered whether Mon was associated with ferroptosis and affected the progression of HCC. The aim of this study was to investigate the possible capacity of Mon to induce ferroptosis in HCC, and to elucidate the underlying mechanisms involved.

## 2 Materials and Methods

### 2.1 Cell Culture and Treatment

Normal human hepatocyte cell line MIHA was obtained from Fenghui Biotech Co. Ltd. (Hunan, China). The HCC cell lines Huh‐7 and SNU‐182 were purchased from American Type Culture Collection (ATCC). The cells were cultured with DMEM or RPMI‐1640 medium at 37°C in a humidified atmosphere with 5% CO_2_. Additionally, the medium was supplemented with 10% fetal bovine serum (FBS) and 100 U/mL of penicillin/streptomycin (Gibco, NY, USA).

Mon (≥ 98% purity), DNA replication inhibitor Mitomycin C (MMC), ferroptosis inducer Erastin, and ferroptosis inhibitor Lip1 were obtained from Sigma‐Aldrich (St Louis, MO, USA). Huh‐7 and SNU‐182 cells were treated with 0, 12.5, 25, and 50 μM Mon for 48 h, respectively. 5 μM of Erastin and 1 μM of Lip1 were added to cells for 2 h before Mon treatment. For MMC treatment, Huh‐7 cells were pre‐treated with 0, 25, 50, and 100 ng/mL of MMC for 2 h, and then fresh culture medium was replaced, followed by 25 μM of Mon treatment.

### 2.2 Cell Transfection

PcDNA 3.1 plasmids encoding nuclear factor E2‐related factor 2 (Nrf2) were utilized to achieve overexpression effects. According to the manufacturer's protocol, lipofectamine 3000 (Invitrogen, CA, USA) was employed to transfect Nrf2‐overexpressed plasmids (OE‐Nrf2) and negative control plasmids (OE‐NC) into Huh‐7 cells, which were further treated with 50 μM of Mon for 12 h post‐transfection.

### 2.3 CCK‐8 Assay

Cell viability was assessed by Cell Counting Kit (CCK)‐8 (Beyotime, Shanghai, China) following the manufacturer's protocol. For each well of the 96‐well plate, 10‐μL CCK‐8 solution was added and incubated at 37°C for 4 h. The absorbance value at 450 nm was detected by a microplate reader (Thermo Fisher Scientific, MA, USA).

### 2.4 Colony Formation Assay

Cell proliferation was evaluated by colony formation assay. Briefly, cells were seeded into 6‐well plates at a density of 400 cells/well. After 10–14 days, the colonies were stained with 0.1% crystal violet solution for 30 min at room temperature, and then the number of colonies in each well was counted.

### 2.5 EdU Assay

Cell proliferation was also evaluated by BeyoClick EdU Cell Proliferation Kit (Beyotime) following the manufacturer's protocol. Briefly, cells were treated with 10 μM of EdU for 2 h to label DNA‐synthesizing cells. After fixation and permeabilization, cells were incubated with the reaction cocktail and the nuclei were stained with DAPI. Then the images were visualized using a fluorescence microscope.

### 2.6 Transwell Assay

The 24‐well Transwell chambers precoated with Matrigel were utilized to evaluate cell invasion. Briefly, the upper chamber was seeded with cell suspension in serum‐free medium, and the lower chamber was supplemented with medium containing 20% FBS. After removing the cells remaining in the upper chamber, the cells on the filter surface were fixed and then stained with crystal violet dye. The images were captured using an optical inverted microscope (magnification = 200×).

### 2.7 Detection of Reactive Oxygen Species (ROS) Production

The intracellular ROS production was detected with 2', 7'‐dichlorofluorescin diacetate (DCFH‐DA) probe (Beyotime). Intracellular ROS can oxidize non‐fluorescent DCFH to generate fluorescent DCF, and the intensity of green fluorescence is proportional to the level of ROS. Briefly, the cells were cultured with DCFH‐DA probe at 37°C for 20 min. A fluorescence microscope was employed to detect fluorescence signals.

### 2.8 Establishment of Animal Model

The animal study was approved by the Animal Care and Use Committee of the First Affiliated Hospital of Hunan University of Chinese Medicine. Male BALB/c nude mice, aged 4–6 weeks, were purchased from the Chengdu DOSSY Experimental Animals Co. Ltd. Briefly, Huh‐7 cells (1 × 10^7^) in 100 μL of PBS were subcutaneously injected into the axilla of each nude mouse. At 2th day, the mice were randomly divided into the Mon group (*n* = 6) and sham group (*n* = 6), which received intraperitoneal injections of 50 μL of Mon (60 mg/kg body weight) or vehicle twice a week for 4 weeks. All the mice were euthanized by cervical dislocation on the 30th day. Then the primary tumors were isolated and weighed, followed by the subsequent experiments.

### 2.9 Detection of MDA, GSH and Fe^2+^ Level

MDA and GSH assay kits were purchased from Beyotime. The MDA and GSH levels in cells or in serum were measured according to the instructions. The absorbance values were detected using a microplate reader. The iron assay kit (Abcam, MA, USA) was used to measure the free ferrous iron (Fe^2+^) levels in cell supernatant or in serum.

### 2.10 Western Blot Assay

Total protein was isolated from cells or tumor tissues by RIPA buffer (Beyotime) in accordance with the protocol. Following protein quantification, the proteins were separated by SDS‐PAGE and subsequently transferred onto nitrocellulose membranes. The membranes were blocked with 5% nonfat milk, followed by incubation with primary antibodies. The membranes were then incubated with horseradish‐peroxidase‐conjugated secondary antibody. Finally, the protein bands were detected by an enhanced chemiluminescence detection kit. The primary antibodies included anti‐E‐cadherin, anti‐N‐cadherin, anti‐Vimentin, anti‐transferrin (TF), anti‐ferroportin (FPN), anti‐ferritin heavy chain 1 (FTH1), anti‐Nrf2, anti‐heme oxygenase‐1 (HO‐1), and anti‐glutathione peroxidase 4 (Gpx‐4).

### 2.11 Immunohistochemical Assay

The primary tumor tissues were fixed and cut into 5‐μm slices. After dehydrating, removing endogenous peroxidase activity, and blocking procedures, the slices were incubated with the primary antibodies (anti‐E‐cadherin, anti‐N‐cadherin, anti‐Vimentin). Then the slices were incubated with biotinylated secondary antibody and visualized with diaminobenzidine (DAB) kit (ZSGB‐BIO, Beijing, China).

### 2.12 Statistical Analysis

GraphPad Prism 8.0 was used to carry out the statistical analysis. Paired *t*‐test was used to analyze the difference between two groups. One‐way analysis of variance followed by Bonferroni's post hoc test was applied to analyze the difference among four or five groups. The data are shown as mean ± standard deviation. *p* < 0.05 was considered a statistically significant difference.

## 3 Results

### 3.1 Mon Exhibited Anti‐Tumor Effects in HCC Cells via Inhibiting Cell Proliferation and Invasion

**FIGURE 1:**
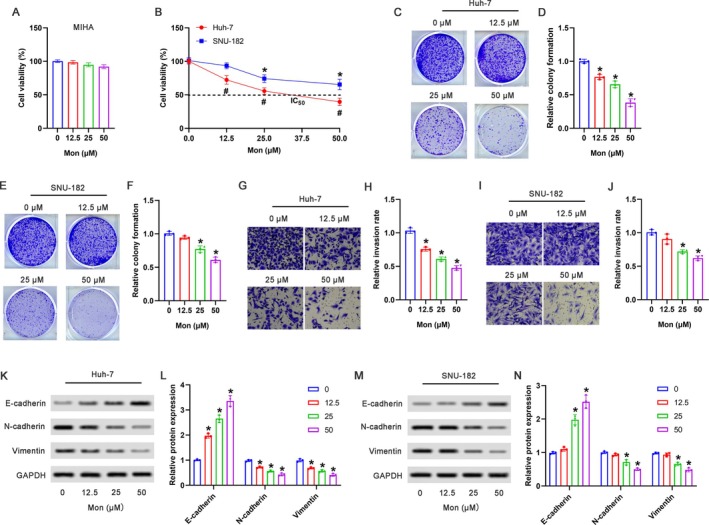
Mon exhibited anti‐tumor effects in HCC cells via inhibiting cell proliferation and cell invasion. MIHA, Huh‐7, and SNU‐182 cells were treated with 0, 12.5, 25, and 50 μM of monotropein for 48 h, respectively. The cell viability of MIHA (A), Huh‐7, and SNU‐182 cells (B) was detected by CCK‐8 assay. (C–D) The representative pictures of colony formation in Huh‐7 cells were shown, and the relative colony formation was analyzed. (E, F) The representative pictures of colony formation in SNU‐182 cells were shown, and the relative colony formation was analyzed. (G, H) The representative pictures of cell invasion in Huh‐7 cells were shown, and the relative invasion rate was analyzed. (I, J) The representative pictures of cell invasion in SNU‐182 cells were shown, and the relative invasion rate was analyzed. (K, L) The representative images of Western blot in Huh‐7 cells were shown, and the relative protein expression of E‐cadherin, N‐cadherin, and Vimentin were analyzed. (M, N) The representative images of Western blot in SNU‐182 cells were shown, and the relative protein expression of E‐cadherin, N‐cadherin, and Vimentin were analyzed. *p < 0.05 versus 0 μM Mon group.

The cytotoxic effects of Mon were determined in normal human hepatocyte cell line MIHA by CCK‐8 assay. As shown in Figure 1A, compared with the 0‐μM Mon group, the cell viability showed no significant decrease in MIHA cells with different doses (12.5, 25, and 50 μM) of Mon treatment. As shown in Figure 1B, compared with the 0‐μM Mon group, the cell viability in the Mon group was suppressed in both Huh‐7 cells and SNU‐182 cells. In Huh‐7 cells, 12.5, 25, and 50 μM Mon induced marked suppression of cell viability. However, in SNU‐182 cells, 25 and 50 μM of Mon treatment but not 12.5 μM induced significant suppression of cell viability, suggesting that Huh‐7 cells had higher sensitivity to the action of Mon than SNU‐182 cells.

Colony formation assay showed that 12.5, 25, and 50 μM of Mon dramatically reduced the colony numbers in Huh‐7 cells compared with the 0‐μM Mon group (Figure 1C,D), while the colony‐forming ability of SNU‐182 cells was effectively inhibited by 25 and 50 μM of Mon but not 12.5 μM (Figure 1E,F). As shown in Figure 1G,H, compared with 0 μM Mon group, 12.5, 25 and 50 μM of Mon signally reduced the cell invasion of Huh‐7 cells. As shown in Figure 1I,J, compared with the 0‐μM Mon group, 25 and 50 μM of Mon but not 12.5 μM observably reduced the cell invasion of SNU‐182 cells. Furthermore, as shown in Figure 1K,L, compared with the 0‐μM Mon group, 12.5, 25, and 50 μM of Mon markedly upregulated the protein expression of E‐cadherin, downregulated the protein expression of N‐cadherin, and Vimentin. As shown in Figure 1M,N, compared with the 0‐μM Mon group, 25 and 50 μM of Mon but not 12.5 μM notably upregulated E‐cadherin expression, as well as downregulated N‐cadherin and Vimentin expression.

Furthermore, we tried to distinguish whether the suppression effect of Mon on cell invasion resulted from its inhibition of cell proliferation, and DNA replication inhibitor MMC was employed to eliminate the confounding factor of increased cell numbers due to proliferation. As shown in Figure S1A, compared with the 0‐h group, the OD_450_ value at 24 and 48‐h groups was significantly increased in the 0‐ng/mL to 2‐h group or the 25‐ng/mL to 2‐h group, respectively; the OD_450_ value in the 50‐ng/mL to 2‐h group showed no significant differences among different times (0, 24, and 48 h); in the 100‐ng/mL to 2‐h group, although the OD_450_ value at 24 h had no obvious reduction compared to that at 0 h, the OD_450_ value at 48 h was significantly decreased, suggesting that the treatment of 50 ng/mL to 2 h in Huh‐7 cells may effectively inhibit cell proliferation without affecting the cell viability of the original cells within the following 48 h. Consistent with that, the cell viability in the 50‐ng/mL to 2‐h group was significantly suppressed at both 24 and 48 h compared to that at 0 h due to the reduced cell number (Figure S1B). Moreover, we verified the cell proliferation by BrdU assay. As shown in Figure S1C, compared with the 0‐ng/mL to 2‐h group, the cell proliferation in the 50‐ng/mL to 2‐h group was markedly suppressed at 24 and 48 h, respectively.

To clarify whether Mon could still inhibit invasion when cell proliferation was suppressed, we pre‐treated Huh‐7 cells with 50 ng/mL of MMC for 2 h, followed by the treatment of 25 μM of Mon. As shown in Figure S1D, compared with the control group, the cell invasion in the MMC group was significantly suppressed, and compared with the MMC group, the cell invasion in the MMC+Mon group was further decreased. Moreover, we detected the expression of EMT protein. As shown in Figure S1E, compared with the control group, the upregulation of E‐cadherin, as well as the downregulation of N‐cadherin and Vimentin in the Mon group showed no significant differences in the presence of MMC pre‐treatment.

### 3.2 Mon Activated Ferroptosis in HCC Cells

**FIGURE 2:**
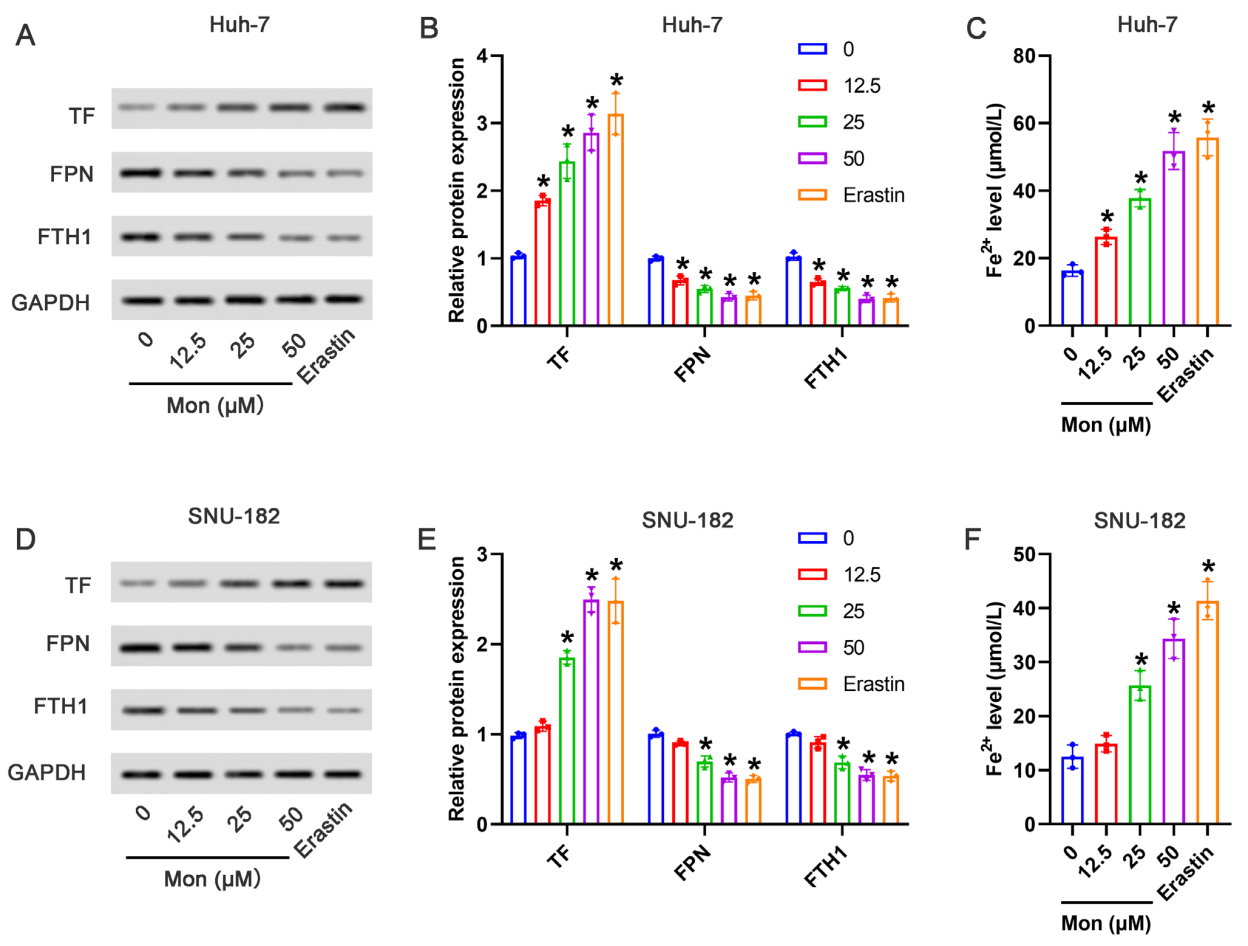
Mon activated ferroptosis in HCC cells. Huh‐7 and SNU‐182 cells were treated with 0, 12.5, 25, 50 μM Mon or 5 μM Erastin, respectively. (A) The representative images of Western blot in Huh‐7 cells were shown. (B) The relative protein expression of TF, FPN, and FTH1 in Huh‐7 cells were analyzed. (C) The Fe^2+^ levels in Huh‐7 cells were detected by the corresponding kit. (D) The representative images of Western blot in SNU‐182 cells were shown. (E) The relative protein expression of TF, FPN, and FTH1 in SNU‐182 cells were analyzed. (F) The Fe^2+^ levels in SNU‐182 cells were detected by the corresponding kit. *p < 0.05 versus 0 μM Mon group.

As shown in Figure 2A,B, compared with the 0‐μM Mon group, 12.5, 25, and 50 μM of Mon significantly increased the protein expression of TF, as well as reduced the protein expression of FPN and FTH1 in Huh‐7 cells. The effects of 50 μM of Mon had no obvious difference with the positive control Erastin. Compared with the 0‐μM Mon group, 12.5, 25, and 50 μM of Mon significantly increased the Fe^2+^ levels in Huh‐7 cells (Figure 2C). Similarly, the effects of Mon in SNU‐182 cells were assessed. As shown in Figure 2D,E, compared with the 0‐μM Mon group, 25 and 50 μM of Mon but not 12.5 μM notably upregulated the protein expression of TF and meanwhile downregulated FPN and FTH1 expression in SNU‐182 cells. As shown in Figure 2F, compared with the 0‐μM Mon group, 25 and 50 μM of Mon but not 12.5 μM markedly increased the Fe^2+^ levels in SNU‐182 cells.

### 3.3 Mon Promoted ROS Accumulation and Lipid Peroxidation in HCC Cells

**FIGURE 3:**
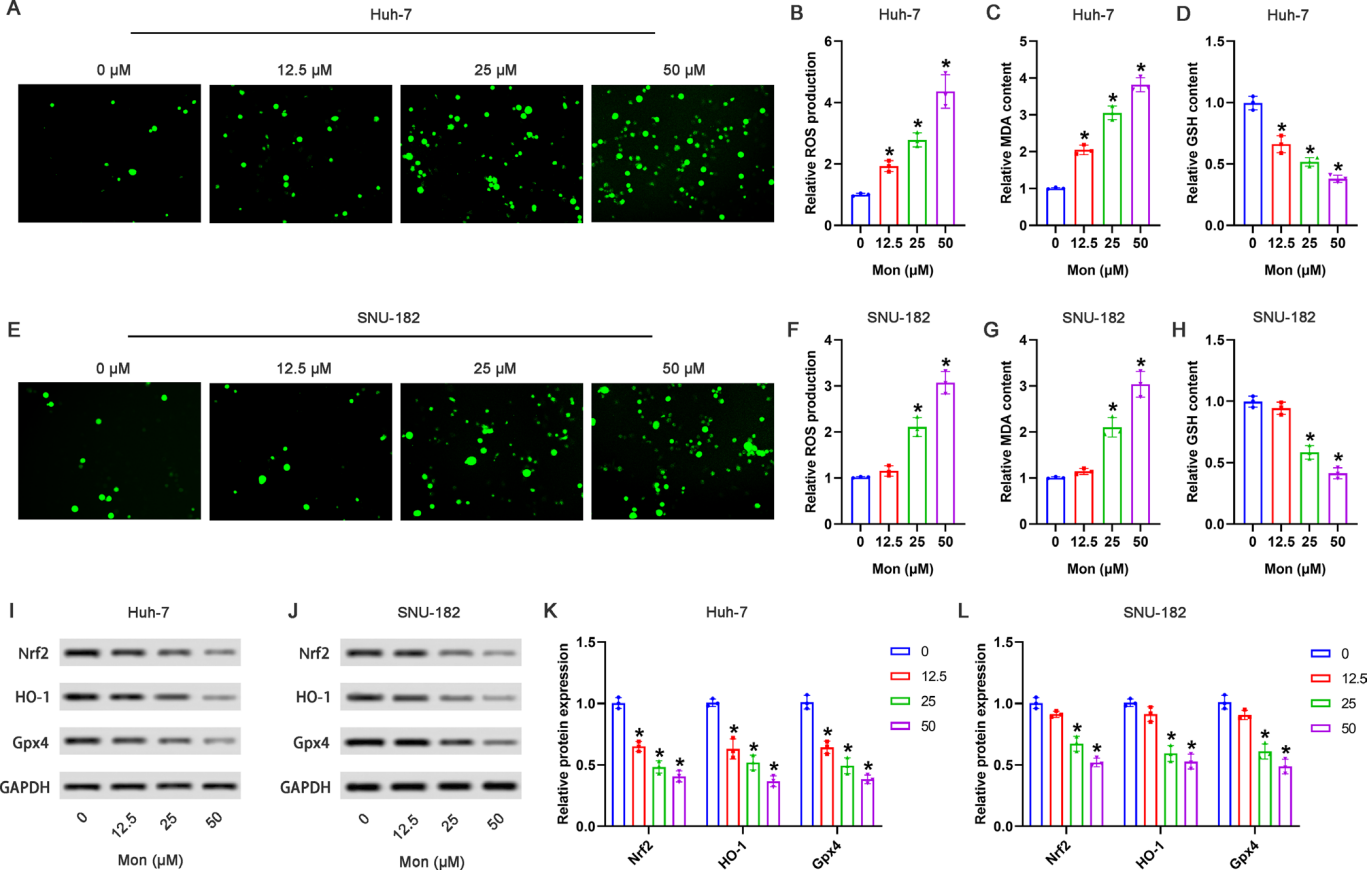
Mon promoted ROS accumulation and lipid peroxidation in HCC cells. Huh‐7 and SNU‐182 cells were treated with 0, 12.5, 25, and 50 μM Mon. (A) The ROS levels in Huh‐7 cells were detected by DCFH‐DA assay and representative images were shown. (B) The relative ROS production in Huh‐7 cells was analyzed. The relative content of MDA (C) and GSH (D) in Huh‐7 cells was detected by corresponding kits. (E) The ROS levels in SNU‐182 cells were detected by DCFH‐DA assay, and the representative images were shown. (F) The relative ROS production in SNU‐182 cells was analyzed. The relative content of MDA (G) and GSH (H) in SNU‐182 cells was detected by corresponding kits. (I, J) The representative images of Western blot were shown. The relative protein expression of Nrf2, HO‐1, and Gpx4 in Huh‐7 cells (K) and SNU‐182 cells (L) was analyzed. *p < 0.05 versus 0 μM Mon group.

As shown in Figure 3A–D, compared with the 0‐μM Mon group, 12.5, 25, and 50 μM of Mon markedly elevated the content of ROS and MDA and reduced the GSH level in Huh‐7 cells. Meanwhile, as shown in Figure 3E–H, compared with the 0‐μM Mon group, 25 and 50 μM of Mon but not 12.5 μM markedly induced the accumulation of ROS and MDA and the reduction of GSH in SNU‐182 cells. The protein expression of the Nrf2 signaling pathway was further measured. Compared with the 0‐μM Mon group, 12.5, 25, and 50 μM of Mon significantly downregulated the protein expression of Nrf2, HO‐1, and Gpx4 in Huh‐7 cells (Figure 3I,K). As shown in Figure 3J,L, compared with the 0‐μM Mon group, 25 and 50 μM of Mon but not 12.5 μM markedly downregulated the protein expression of Nrf2, HO‐1, and Gpx4 in SNU‐182 cells.

### 3.4 Mon Induced Ferroptosis via the Inhibition of Nrf2 Signaling in HCC Cells

**FIGURE 4:**
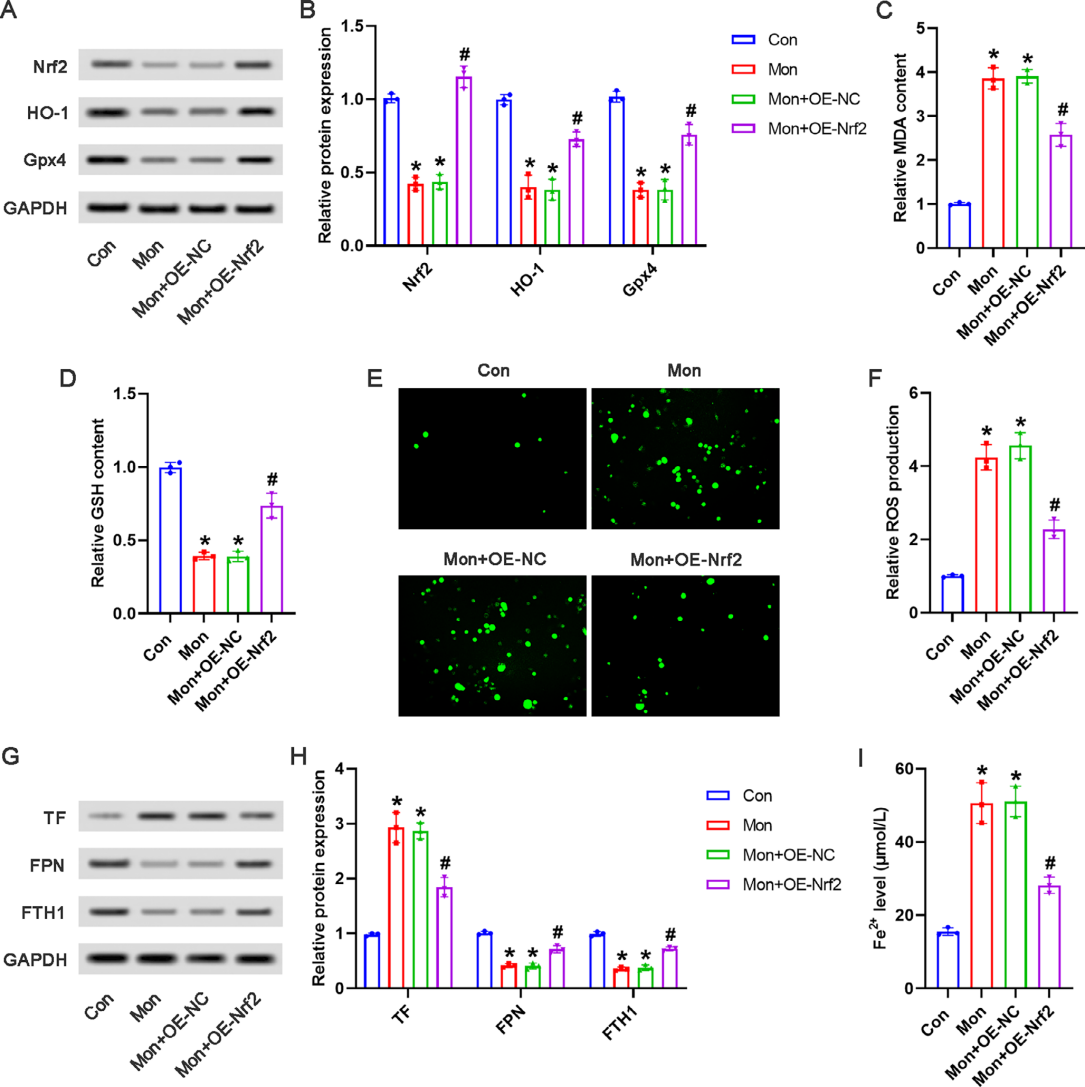
Mon induced ferroptosis via the inhibition of Nrf2 signaling in HCC cells. Huh‐7 cells were transfected with Nrf2‐overexpressed plasmids or negative empty plasmids, followed by treatment with 50 μM of Mon. (A) The representative images of Western blot were shown. (B) The relative protein expression of Nrf2, HO‐1, and Gpx4 was analyzed. The relative content of MDA (C) and GSH (D) was detected by corresponding kits. (E) The ROS levels were detected by DCFH‐DA assay, and the representative images were shown. (F) The relative ROS production was analyzed. (G) The representative images of Western blot were shown. (H) The relative protein expression of TF, FPN, and FTH1 was analyzed. (I) The Fe^2+^ levels were detected by the corresponding kit. *p < 0.05 versus Con group; #*p* < 0.05 versus Mon+OE‐NC group.

As shown in Figure 4A,B, Mon actually downregulated the Nrf2, HO‐1, and Gpx4 expression in Huh‐7 cells; compared with the Mon+OE‐NC group, the downregulated expression of Nrf2, HO‐1, and Gpx4 was observably reversed by Nrf2 overexpression. Furthermore, the Mon‐stimulated elevated MDA and reduced GSH in Huh‐7 cells had been partially reversed by Nrf2 overexpression (Figure 4C,D). As shown in Figure 4E,F, compared with the Con group, the ROS production was significantly increased in the Mon group or the Mon+OE‐NC group, which showed an obvious reduction in the Mon+OE‐Nrf2 group. Meanwhile, the upregulated TF expression and downregulated FPN and FTH1 expression in the Mon+OE‐NC group were at least in part reversed in the Mon+OE‐Nrf2 group (Figure 4G,H). In addition, as shown in Figure 4I, the increased Fe^2+^ level in the Mon+OE‐NC group was also notably inhibited in the Mon+OE‐Nrf2 group.

### 3.5 Mon Exhibited Anti‐Tumor Effects via Inducing Ferroptosis in HCC Cells

**FIGURE 5:**
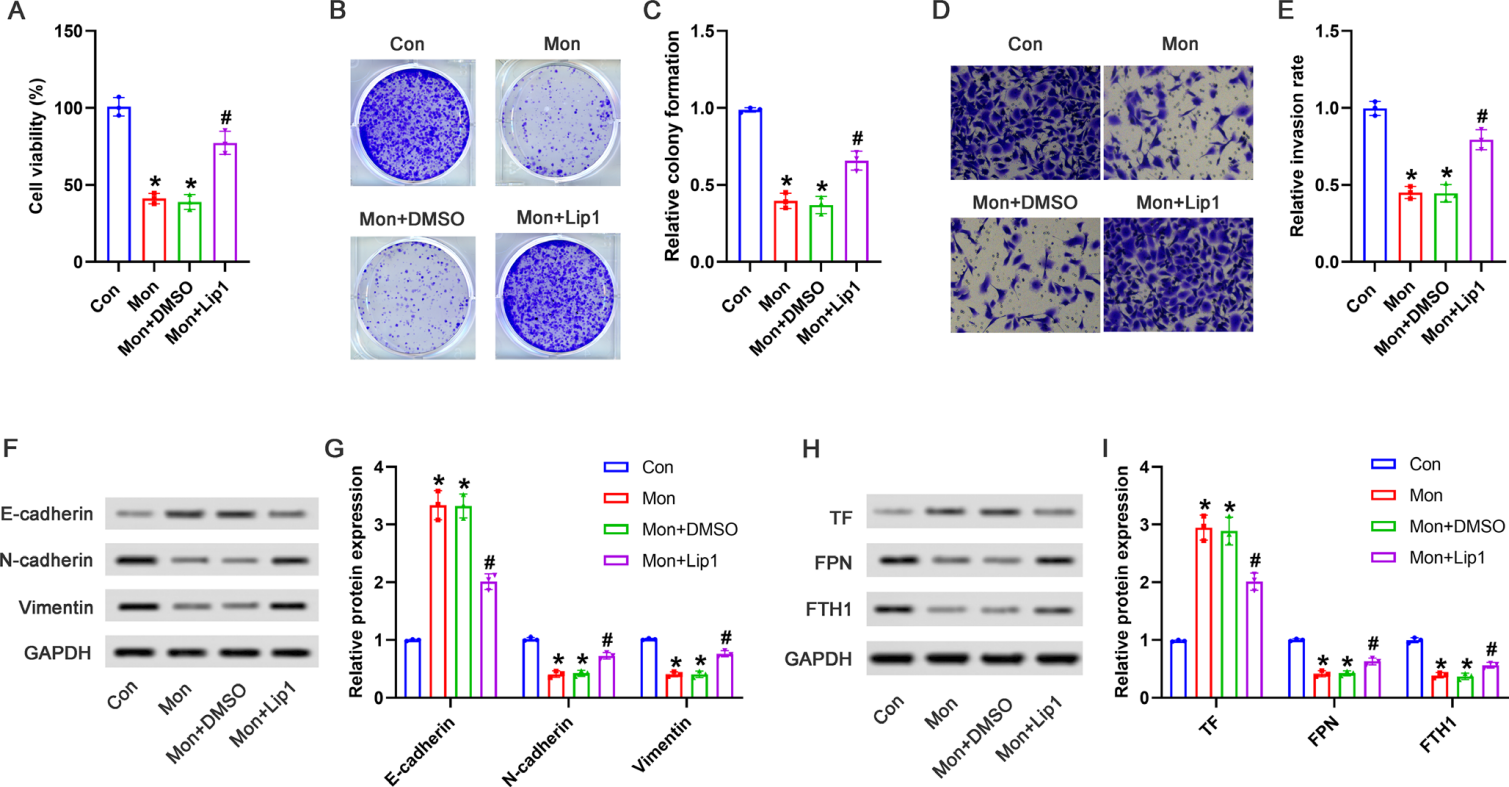
Mon exhibited anti‐tumor effects via inducing ferroptosis in HCC cells. Huh‐7 cells were treated with 1 μM of Lip1, followed by treatment with 50 μM of Mon. (A) The cell viability was detected by the CCK‐8 assay. (B) The representative pictures of colony formation were shown. (C) The relative colony formation was analyzed. (D) The representative pictures of cell invasion were shown. (E) The relative invasion rates were analyzed. (F) The representative images of Western blot were shown. (G) The relative protein expression of E‐cadherin, N‐cadherin, and Vimentin was analyzed. (H) The representative images of Western blot were shown. (I) The relative protein expression of TF, FPN, and FTH1 was analyzed. *p < 0.05 versus Con group; #*p* < 0.05 versus Mon+DMSO group.

As shown in Figure 5A, compared with the Con group, the cell viability was notably suppressed in the Mon group or the Mon+DMSO group; compared with the Mon+DMSO group, the cell viability demonstrated a significant elevation in the Mon+Lip1 group. As shown in Figure 5B,C, compared with the Mon+DMSO group, the reduced colony numbers induced by Mon treatment in Huh‐7 cells showed a significant increase in the Mon+Lip1 group. As shown in Figure 5D,E, the reduced cell invasion of Huh‐7 cells induced by Mon treatment exhibited a marked increase in the Mon+Lip1 group. Moreover, the upregulated E‐cadherin expression, as well as the downregulated N‐cadherin and Vimentin expression in the Mon+DMSO group was significantly reversed by Lip1 treatment (Figure 5F,G). In addition, the upregulated TF expression and downregulated FPN and FTH1 expression in the Mon+DMSO group were partially reversed in the Mon+Lip1 group (Figure 5H,I).

### 3.6 Mon Induced Ferroptosis to Suppress the Progression of HCC

**FIGURE 6:**
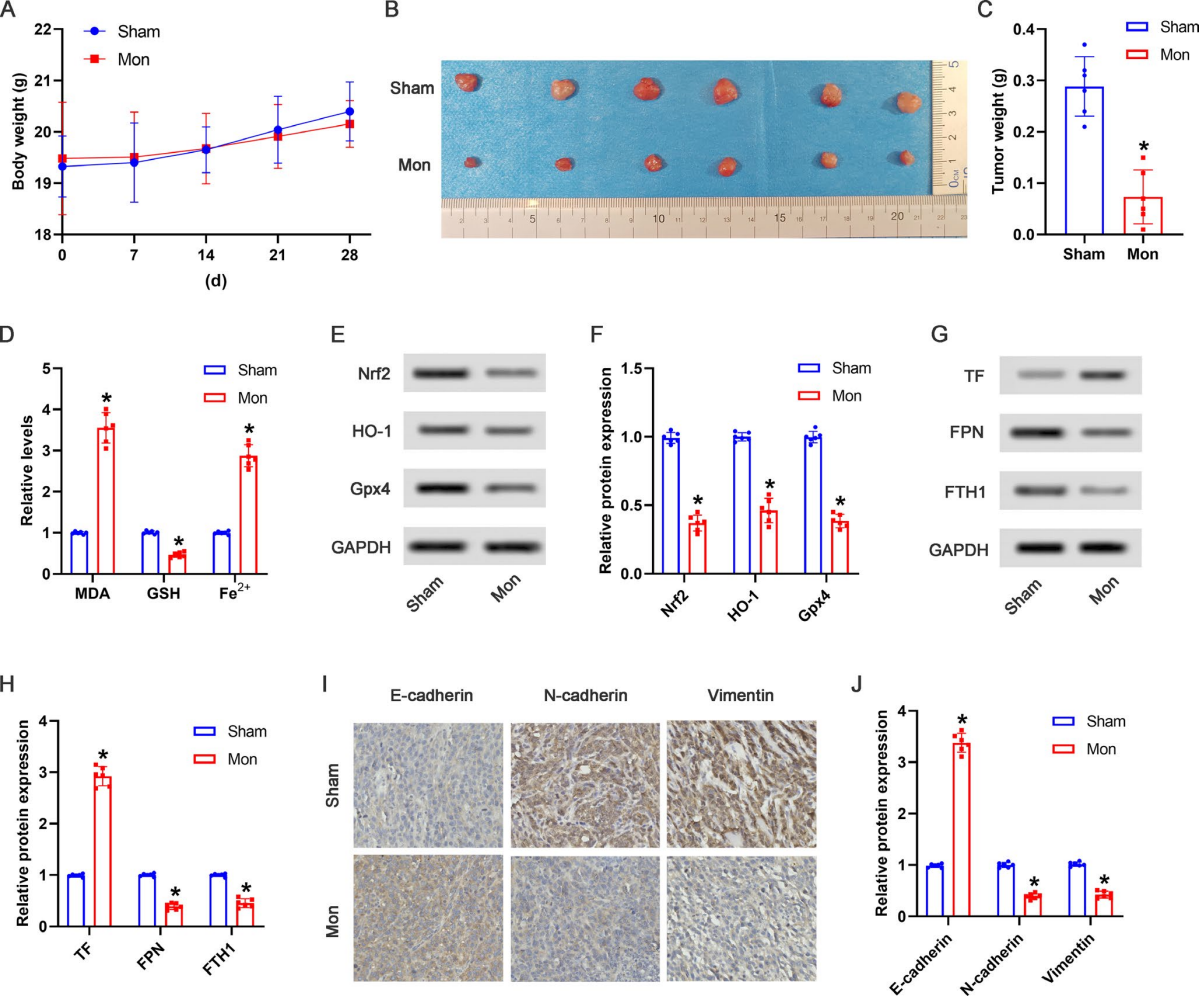
Mon induced ferroptosis to suppress the progression of HCC. Xenograft mouse model was established by the subcutaneous injection of Huh‐7 cells to nude mouse, followed by intraperitoneal injection of Mon. (A) Body weights of the mice were detected during the experiment. (B) Primary tumors from each group were photographed after sacrifice at the 30th day. (C) Tumor weights were measured after sacrifice at the 30th day. (D) The relative levels of MDA, GSH, and Fe^2+^ in serum were detected by corresponding kits. (E) The representative bands of Nrf2, HO‐1, and Gpx4 in primary tumor tissues were shown. (F) The relative protein expression of Nrf2, HO‐1, and Gpx4 was analyzed. (G) The representative bands of TF, FPN, and FTH1 in primary tumor tissues were shown. (H) The relative protein expression of TF, FPN, and FTH1 was analyzed. (I) The expression of E‐cadherin, N‐cadherin, and Vimentin in primary tumor tissues was visualized by immunohistochemistry assay. (J) The relative protein expression of E‐cadherin, N‐cadherin, and Vimentin was analyzed. *p < 0.05 versus Sham group.

As shown in Figure 6A, the body weights of the mice were not significantly influenced by Mon treatment. As shown in Figure 6B,C, the primary tumor weights were significantly reduced in the Mon group compared to the sham group. As shown in Figure 6D, compared with the sham group, the MDA and Fe^2+^ levels in serum were markedly increased, and the GSH content showed a significant reduction in the Mon group. Furthermore, the expression of Nrf2, HO‐1, and Gpx4 protein in primary tumor tissues was downregulated by the Mon treatment (Figure 6E,F). As shown in Figure 6G,H, compared with the sham group, the upregulated protein expression of TF and the downregulated FPN and FTH1 expressions were found in the Mon group. IHC assay showed that compared with the sham group, the E‐cadherin expression was observably upregulated, and the N‐cadherin and Vimentin expressions were notably downregulated in the Mon group (Figure 6I,J).

## 4 Discussion

Although multiple bioactivities of Mon have been reported in various diseases, the biological roles of Mon on HCC and the underlying regulating mechanisms require further investigation. In the current study, we explored the effects of Mon in vitro (Huh‐7 and SNU‐182 cells) and in vivo. Our results demonstrated that Mon exerted its anti‐tumor activity by inhibiting cell proliferation and invasion in HCC cells. Subsequently, we demonstrated that Mon induced ferroptosis characterized by the enrichment of intracellular Fe^2+^ and dysregulation of iron metabolism‐related proteins, accompanied by the accumulation of ROS and the peroxidation of lipid in vitro and in vivo.

The uncontrolled proliferation and invasive potential of tumor cells are key drivers of tumor progression. Therefore, blocking cell proliferation and cell invasion are effective therapeutic strategies for tumors. In the current study, we first demonstrated that Mon treatment had no significant effect on the cell viability of MIHA cells, but markedly suppressed the cell viability of HCC cells, indicating that Mon might play an anti‐tumor role with minimal cytotoxicity. Furthermore, we verified that Mon suppressed both cell proliferation and invasion of HCC cells, with Huh‐7 cells exhibiting greater sensitivity to the action of Mon than SNU‐182 cells. Epithelial‐to‐mesenchymal transition (EMT) is a key process contributing to the invasive capabilities of tumor cells and promotes metastasis [22]. The aberrant activation of EMT can lead to enhanced invasiveness and increased malignancy, eventually resulting in worse prognosis [23, 24]. Furthermore, we detected the expression of EMT‐related proteins. The results showed that Mon elevated E‐cadherin expression while reducing N‐cadherin and Vimentin levels, suggesting that the inhibition of cell invasion by Mon was at least in part associated with its suppression of EMT.

Furthermore, DNA replication inhibitor MMC was employed to distinguish whether the suppression effect of Mon on cell invasion and EMT resulted from its inhibition of cell proliferation. The results showed that the treatment of 50 ng/mL to 2 h of MMC in Huh‐7 cells might effectively suppress the increase of cell number due to the inhibition of cell proliferation, without affecting the cell viability of the original cells, demonstrated by the reduced cell viability and the stable OD_450_ value at 24 and 48 h compared to that at 0 h. EdU assay verified the proliferation‐inhibition effect of 50 ng/mL to 2 h of MMC. Therefore, 50 ng/mL to 2 h of MMC was chosen to clarify whether Mon could still inhibit invasion when cell proliferation was suppressed in Huh‐7 cells.

In the present study, the reduced cell invasion in the MMC group indicated that the invasive capability of Huh‐7 cells was at least in part dependent on its proliferation; therefore, the suppression of cell invasion in the Mon group may be associated with its capability to suppress cell proliferation (in a proliferation‐dependent manner). Compared to the MMC group, the cell invasion in the MMC+Mon group was further decreased, indicating that Mon had a direct action on invasion suppression (in a proliferation‐independent manner) besides in a proliferation‐dependent manner. Additionally, the upregulation of E‐cadherin, as well as the downregulation of N‐cadherin and Vimentin induced by Mon showed no significant differences whether cell proliferation was pre‐inhibited or not, confirming that Mon could directly suppress cell invasion by modulating EMT. Together, the suppression effect of Mon on cell invasion was not only related to its capability to inhibit proliferation (indirect suppression in a proliferation‐dependent manner) but also could directly suppress cell invasion by regulating EMT proteins (direct suppression in a proliferation‐independent manner).

Currently, the induction of ferroptosis as a therapeutic strategy is gaining increasing interest due to its reliance on iron accumulation. The regulation of iron was closely associated with iron metabolism‐related proteins. In the context of ferroptosis, TF and its receptor lead to a well‐organized performance of iron uptake [25]. FPN is a transmembrane protein that serves as the sole exporter of cellular iron in vertebrate species [26]. FTH1, the heavy subunit of ferritin, plays an important role in governing cellular iron concentrations by acting as a reservoir for intracellular iron [27]. In the present study, Mon significantly upregulated TF expression while downregulating FPN and FTH1 expression in Huh‐7 and SNU‐182 cells, indicating that Mon induces an accumulation of iron. The detection of Fe^2+^ level further corroborated this hypothesis.

Specifically, the toxicity of free iron, depletion of the antioxidant GSH, and accumulation of lipid peroxidation are essential conditions for ferroptosis. Elevated oxygen radicals, particularly stemming from an excess of intracellular iron, foster the buildup of lipid peroxidation products, wherein MDA is a prominent component. The accumulation of MDA resulting from lipid peroxidation serves as a pivotal factor in advancing the progression of ferroptosis [28, 29]. GSH is a tripeptide composed of three amino acids that plays a vital role in preventing oxidation, regenerating antioxidants, and maintaining cellular redox balance [30]. Moreover, the abundance of GSH is a substrate for Gpx4, a selenocysteine‐containing enzyme that protects cells from oxidative damage by suppressing the formation of lipid peroxides [31]. Therefore, GSH abundance is preventive against ferroptosis via the reduction of lipid peroxidation. In the current study, we further measured the content of ROS, MDA, and GSH. The results demonstrated that Mon elevated the production of ROS and MDA; meanwhile, Mon decreased the content of GSH, indicating that Mon induced the accumulation of iron‐dependent lipid peroxidation, which accelerated ferroptosis in HCC cells.

We have confirmed that Mon induced ferroptosis characterized by iron accumulation and lipid peroxidation in HCC cells. We further explored the underlying mechanism of Mon regulating ferroptosis. Ferroptosis is closely associated with the regulation of the antioxidant pathway. NRF2, a well‐known transcription factor crucial for antioxidative processes, regulates a variety of genes essential for ferroptosis, such as HO‐1 and Gpx4 [32, 33]. Liu et al. [34] have demonstrated that Wogonin promotes ferroptosis via suppressing the Nrf2/Gpx4 axis in pancreatic cancer cells. Gao et al. [35] have found that lysionotin suppresses colorectal cancer progression by inducing ferroptosis via attenuating Nrf2 signaling. Therefore, we investigated whether the effects of Mon in HCC cells were associated with Nrf2 signaling. In the current study, Mon observably suppressed the protein expression of Nrf2, HO‐1, and Gpx4 in HCC cells, implying that the suppression of Nrf2 signaling may be closely associated with the pro‐ferroptosis role of Mon. To further validate this hypothesis, we employed plasmids overexpressing Nrf2. When Nrf2 was overexpressed, Mon‐induced ferroptosis was markedly suppressed, including the upregulated HO‐1 and Gpx4 expression, reduced ROS and MDA production, elevated GSH content, as well as decreased Fe^2+^ levels. The above data indicated that Mon induced ferroptosis via the inhibition of Nrf2 signaling in HCC cells.

Because the induction of ferroptosis has been recognized as a crucial strategy for cancer treatment, we further validated that the anti‐tumor effects of Mon in vitro were at least partially attributed to the ferroptosis activation by using Lip1. Furthermore, we investigated the effects of Mon in an in vivo tumorigenicity experiment. Consistent with our in vitro findings, Mon suppressed the progression of HCC by inducing ferroptosis in vivo with fewer side effects.

In conclusion, the present findings demonstrated Mon suppressed the progression of HCC both in vitro and in vivo, which was closely associated with the induction of ferroptosis via inhibiting Nrf2 signaling. In the context of ferroptosis‐mediated tumor therapy, the present study indicated that Mon represented a promising alternative for HCC treatment, warranting further investigation into the therapeutic potential of natural compounds.

## Conflicts of Interest

The authors declare no conflicts of interest.

## Supporting information


**Figure S1.** Mon exhibited anti‐invasive effects in Huh‐7 cells in both proliferation‐dependent and proliferation‐independent manners. Huh‐7 cells were pre‐treated with 0, 25, 50, and 100 ng/mL of MMC for 2 h and then fresh culture medium was replaced, followed by 25 μM Mon treatment for 24 or 48 h. The OD_450_ value (A) and cell viability (B) were assessed by CCK‐8 assay. *p < 0.05 versus 0 h or 0 ng/mL of MMC‐2 h group. (C) The cell proliferation was detected by EdU assay. *p < 0.05 versus 0 ng/mL of MMC‐2 h group. (D) The representative pictures of cell invasion were shown, and the relative invasion rate was analyzed. *p < 0.05 versus control group, #*p* < 0.05 versus 50 ng/mL of MMC‐2 h group. (E) The representative images of Western blot were shown, and the relative protein expression of E‐cadherin, N‐cadherin, and Vimentin was analyzed. *p < 0.05 versus control group.

## Data Availability

The data used and analyzed during the current study are available from the corresponding author upon reasonable request.
