# Supplementary figures and images for: Monotropein Induced Ferroptosis to Alleviate the Progression of Hepatocellular Carcinoma via Regulating Nrf2/HO‐1/GPX4 Axis
Source: Kaohsiung J Med Sci. 2025 May 29;41(8):e70034. doi: 10.1002/kjm2.70034 (PMC12407331; doi:10.1002/kjm2.70034)

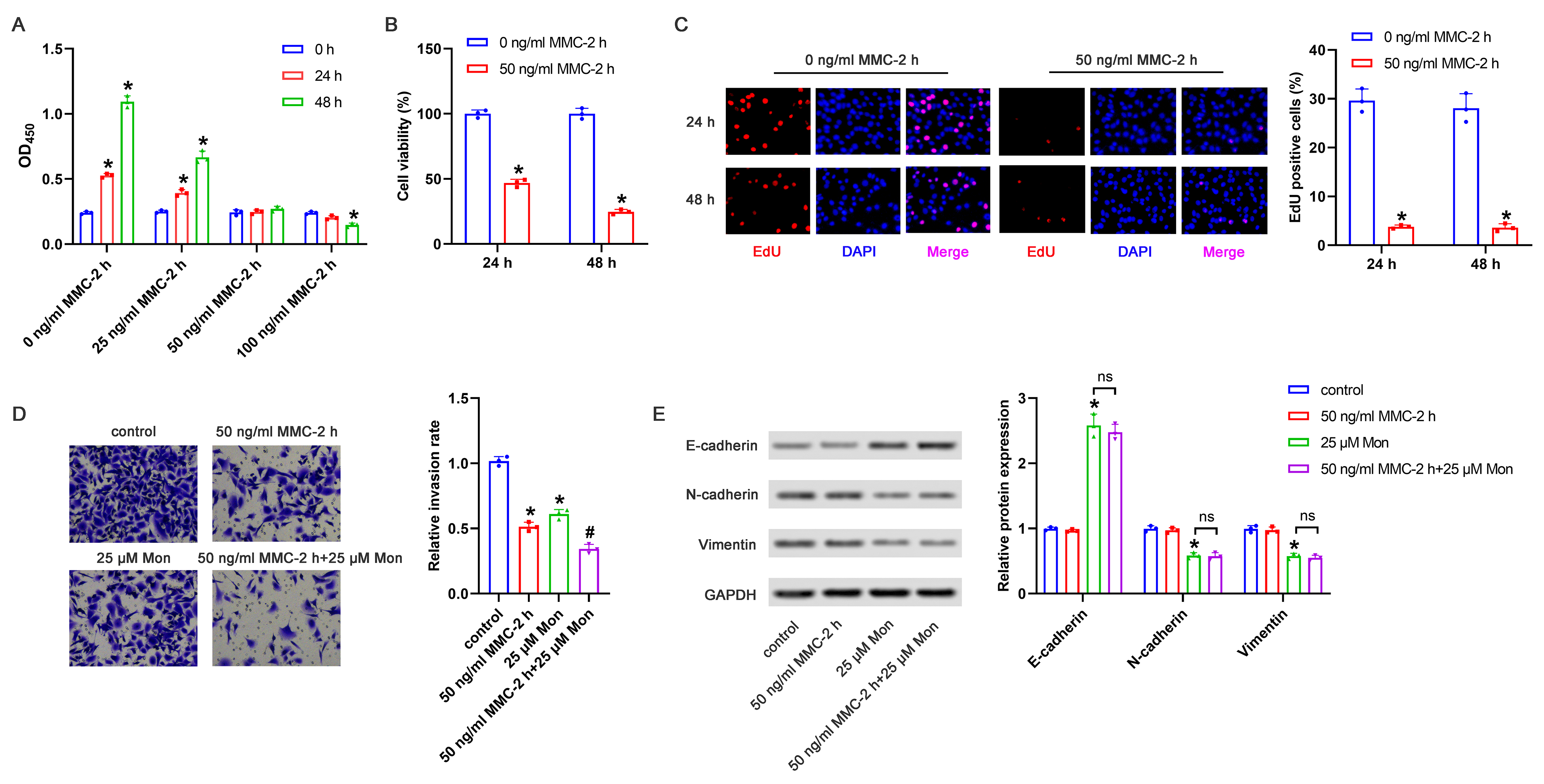

Supplement: Supplementary file 1 — Figure S1. Mon exhibited anti‐invasive effects in Huh‐7 cells in both proliferation‐dependent and proliferation‐independent manners. Huh‐7 cells were pre‐treated with 0, 25, 50, and 100 ng/mL of MMC for 2 h and then fresh culture medium was replaced, followed by 25 μM Mon treatment for 24 or 48 h. The OD450 value (A) and cell viability (B) were assessed by CCK‐8 assay. *p < 0.05 versus 0 h or 0 ng/mL of MMC‐2 h group. (C) The cell proliferation was detected by EdU assay. *p < 0.05 versus 0 ng/mL of MMC‐2 h group. (D) The representative pictures of cell invasion were shown, and the relative invasion rate was analyzed. *p < 0.05 versus control group, #p < 0.05 versus 50 ng/mL of MMC‐2 h group. (E) The representative images of Western blot were shown, and the relative protein expression of E‐cadherin, N‐cadherin, and Vimentin was analyzed. *p < 0.05 versus control group. [file KJM2-41-e70034-s001.tif]
